# Adverse perinatal conditions and receiving a disability pension early in life

**DOI:** 10.1371/journal.pone.0229285

**Published:** 2020-02-24

**Authors:** Fredinah Namatovu, Erling Häggström Lundevaller, Lotta Vikström, Nawi Ng

**Affiliations:** 1 Centre for Demographic and Ageing Research (CEDAR), Umeå University, Umeå, Sweden; 2 Umeå School of Business, Economics, and Statistics, Umeå University, Umeå, Sweden; 3 Department of Historical, Philosophical and Religious Studies, Centre for Demographic and Ageing Research (CEDAR), Umeå University, Umeå, Sweden; 4 Department of Epidemiology and Global Health, Faculty of Medicine, Umeå University, Umeå, Sweden; 5 Global and Public Health, School of Public Health and Community Medicine, Institute of Medicine, Sahlgrenska Academy, University of Gothenburg, Gothenburg, Sweden; University of New South Wales, AUSTRALIA

## Abstract

**Objective:**

The number of young adults on disability pension (DP) is increasing in European countries, creating a need to understand the related risk factors. This study aimed to determine whether adverse perinatal conditions are associated with receiving a DP early in life.

**Methods:**

This longitudinal cohort study consisted of all persons (N = 453,223) born in Sweden during 1973–1977, observed from 1991 through 2010 when they were aged between 16 and 37 years. Statistics Sweden provided linked national data on the children and their parents. We used logistic regression to assess the association between perinatal health conditions (birth defect, Apgar score, and small for gestational age) and receiving a DP, adjusting for maternal education and the sex of the child.

**Results:**

New recipients of DP were significantly more likely to have had a birth defect (adjusted odds ratio [AOR] 2.74, 95% CI: 2.49–3.00), to have had low Apgar score (AOR 2.12, 95% CI: 1.77–2.52), to have been small for gestational age (AOR 1.73, 95% CI: 1.54–1.94) and to be females (AOR 1.55, 95% CI: 1.46–1.64). Higher maternal education was associated with lower odds of receiving a DP (AOR 0.74, 95% CI: 0.69–0.79) for those with high school education and (AOR 0.67, 95% CI: 0.59–0.75) for those with university education. Age-stratified analysis confirmed increased odds of receiving a DP among those with birth defects and small for gestational age, but this effect reduced with increasing age. Apgar score was significantly associated with starting to receive a DP at ages 16–18 and 19–29, but not at ages 30–33. Women had lower odds of receiving a DP at ages 16–18 (AOR 0.73, 95% CI: 0.64–0.85); however, this reversed from age 19 and upwards (AOR 1.53, 95% CI: 1.41–1.67) and (AOR 2.16, 95% CI: 1.95–2.40) for the age groups of 19–29 and 30–33, respectively. Persons with high maternal education were less likely to receive a DP regardless of age at receiving a DP.

**Conclusion:**

Having a birth defect was the strongest indicator of receiving a DP during early adulthood, followed by small for gestational age and low Apgar score. Overall, the effects of the studied perinatal health conditions were pronounced in those who received a DP at 16–18 years, but this effect weakened with increasing age at receiving a DP. Our findings suggest that policies and programs geared at promoting optimal health at birth might contribute to a reduction in receiving a DP.

## Introduction

Over the past few decades, Europe has witnessed an increase in the number of people receiving a disability pension (DP), [1, 2] with several countries reporting a high proportion of young adults as new recipients. [2–4] This trend of early exit from the labor force and reliance on DP increases financial pressures on governments and aggravates the anticipated future labor force shortage due to the aging population. [2] DP is a social security scheme that provides income support to people of working age with long-term limitations in their working capacity due to ill health, and it is an important part of the public support programs for people with disabilities in Sweden. [3, 4]

Several studies have identified numerous socio-economic and health-related factors associated with the utilization of DP. [2, 5] Some of the identified adulthood socio-demographic risk factors include education, occupation, marital status, family structure, and place of residence.[6–12] Individuals receiving DP also tend to report more adverse health outcomes, poorer self-rated health, increased alcohol use, and more frequent use of primary health care. [7, 10, 13–15]

A few studies using the life course critical model have examined the link between childhood conditions and the risk of receiving a DP later in life [16]. The critical model suggests that suboptimal perinatal conditions cause long-lasting changes in the developing organ structures and in the functioning of biological systems, which in turn places an individual at an increased risk of chronic diseases during adulthood. [16] Furthermore, a handful of studies have established a link between childhood socio-economic position and the risk of having a disability later in life. [17, 18] Some studies noted that receiving a DP during adulthood was higher among persons with low birth weight [19, 20] and among those born small for gestational age. [11] However, the evidence on the linkage between perinatal health and the receipt of a DP during early adulthood is still insufficient for drawing any firm conclusions.

From our literature search, we identified no single study that has investigated the association between having a birth defect or a low Apgar score and receiving a DP during early adulthood. Some evidence does suggest that persons with birth defects are more likely to report a developmental disability later in life [21–23], and another study reported a link between low Apgar score at five minutes and minor disabilities at school age. [24] Low Apgar score was also associated with having a neurological disability in early adulthood. [25, 26]

Factors associated with an increased risk of any form of disability are also like to affect one’s overall health, and as such are likely to increase work incapacity that leads to receiving a DP. Thus, we hypothesized that having a birth defect or a low Apgar score is associated with receiving a DP in early adulthood. To test this hypothesis, we followed the birth cohort of 1973–1977 from ages 16 to 37. The aims of this study were (a) to investigate the association between perinatal health factors as measured by birth defects, Apgar score, and being small for gestational age and the receipt of a DP during early adulthood, and (b) to assess whether the effect of these perinatal outcomes differ according to the age at which one starts to receive a DP.

## Material and methods

### Study design and study population

**Fig 1:**
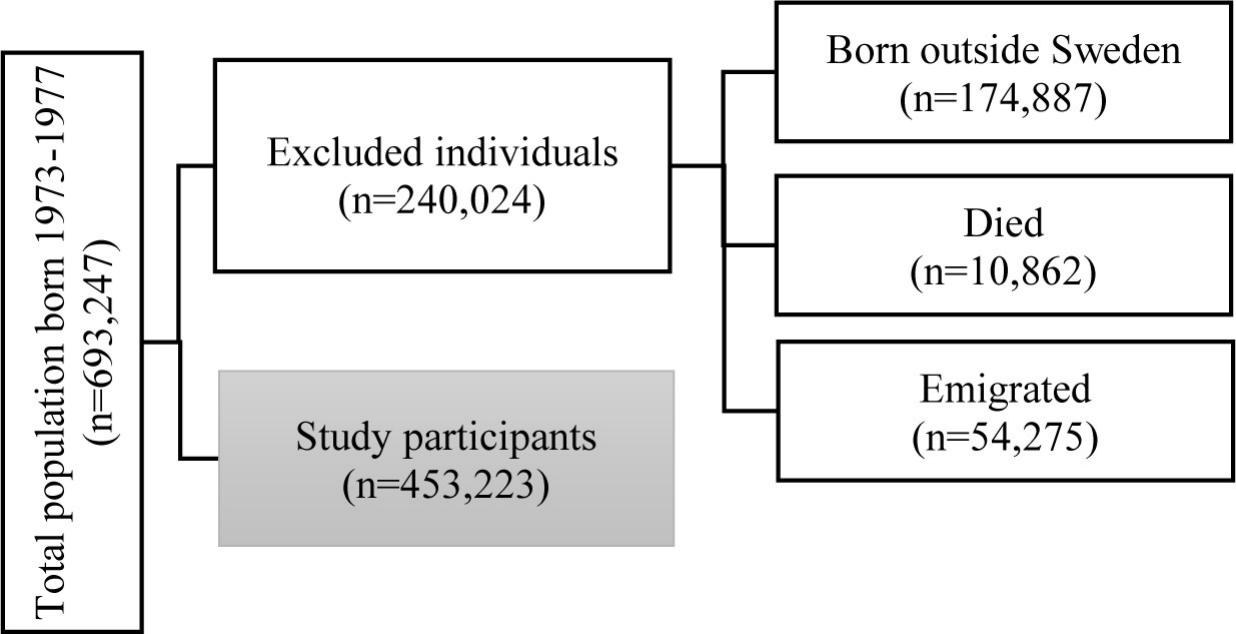
Selection criteria for the study participants.

In this longitudinal cohort study, we used national register data that consisted of all persons born in Sweden from 1973 to 1977. We followed these individuals from 1990 up to 2010 when they were between 16 and 37 years of age. Initially, we identified 693,247 persons. We excluded 240,024 persons because they were born outside of Sweden, had died, or had emigrated. We restricted our analysis to 453,223 individuals who met the study criteria of being born in Sweden and living in Sweden during the observation period (Fig 1).

The Swedish Medical Birth Register provided information on the total newborn population and their perinatal health outcomes, including information on birth defects (congenital anomalies at birth), Apgar score and small for gestation age. [27] We obtained information on sex, mother’s education level, and receipt of disability benefits from the Longitudinal Integration Database for Health Insurance and Labour Market Studies (LISA database). Statistics Sweden linked the index person’s data and the data of their mother obtained from these two data sources using their unique Swedish personal identity numbers. After data linkage, Statistics Sweden made the data anonymous and delivered it to the Swedish Initiative for Research on Microdata in Social and Medical Sciences (Umeå SIMSAM Lab), [28] where we performed all analyses.

### Study variables

*Receiving a DP*: This outcome variable was measured when the individuals were between the ages of 16 and 37. Sweden uses a systematic medical examination, as codified by Swedish social security legislation, to measure diminished health and work capacity when assessing eligibility to receive this financial benefit. [3–5] However, Swedish DP legislation has frequently changed. From 1991–2002, individuals were eligible to receive a DP if they were between the ages of 16 and 64 with medical evidence confirming their inability to work due to chronic ill health. [3, 4] From 2003 onwards, the medical basis for granting this financial security remained the same, but the minimum age limit was raised to age 19 and the term DP was replaced with activity compensation, which is payable to persons aged 19–29, and sickness compensation, which is payable to persons aged 30–64. In this paper, we use the term DP as an umbrella term that includes DP, activity compensation, and sickness compensation. We recorded individuals as having received a DP from their first year of receiving the benefit, “yes” for those who received a DP and “no” for those who did not. We further created the age groups of 16–18, 19–29, and 30–33 years to determine if the effect differed by the age of receiving a DP. These categories were predetermined to reflect the age consideration in the DP legislation during the follow-up period. Persons aged 16–18 years were only eligible for DP prior to the 2002 legislation.

When selecting explanatory variables, we included perinatal health variables because previous research suggests a link [19–23] and/or because we considered them as plausible risk factors for receiving a DP. We obtained the variable birth defect from the Medical Birth Register, already coded in the register as yes or no. The variable Apgar score at 5 minutes measures the physical condition of the newborn at 5 minutes after birth on a scale of 0–10. [29] We categorized this variable according to existing criteria, which considers a total score of less than 7 as a low Apgar score, while a score within a range of 7 to 10 is considered normal. [29, 30] Gestational age was pre-categorized into two groups based on the Swedish growth standards that account for both birth weight for gestational age and sex. The 5^th^ percentile (z-score below −1.64) was the threshold, and individuals below this were pre-categorized as small for gestational age, and those above were considered appropriate for gestational age. We categorized sex as either man or woman, and maternal education was categorized into ≤9 years of school (reference category), upper secondary education and university education.

### Statistical analyses

In the descriptive analysis, we used cross tabulation to compare the explanatory variables between individuals who received a DP and those who did not (Table 1). We checked for multi-collinearity between all independent variables and found no evidence for this. We conducted logistic regression to determine whether any of the perinatal conditions were associated with the odds of receiving a DP between the ages of 16 and 37. We examined the independent association between each of the variables and the odds of receiving a DP (Table 2). In Models 1–3 in Table 2, we tested changes in the odds of receiving a DP by adjusting for different sets of covariates. In Model 1, we included all perinatal health variables, and in Model 2 we added sex and maternal education to Model 1. In Model 3 we considered all variables included in Model 2 plus all possible interactions between explanatory variables i.e., the interaction between birth defect and sex, sex and Apgar scores, sex and gestation age, mother’s education and sex, birth defect and Apgar score, birth defect and small for gestation age, and small for gestational age and Apgar score.

**Table 1:** Perinatal characteristics and bivariate results of the 1973–1977 birth cohort by DP status (n = 453,223).

Description	No Disability Pension N = 434,369 (96%)	(%)	Disability Pension N = 18,854 (4%)	%	Bivariate results OR (95% CI) p-value
**Birth defect**					
No	411,498	(96)	16,939	(4)	1.00
Yes	19,523	(92)	1,739	(8)	2.16 (2.05–2.28) ***
Data missing	3,348	(95)	176	(5)	
**Apgar at 5 minutes**					
≥7	311,306	(96)	13,159	(4)	1.00
<7	4,406	(91)	428	(9)	2.29 (2.08–2.54) ***
Data missing	118,657	(96)	5,267	(4)	
**Small for gestational age**					
No	403,644	(96)	16,767	(4)	1.00
Yes	18,516	(93)	1,422	(7)	1.85 (1.75–1.95)***
Data missing	12,209	(95)	665	(5)	
**Sex**					
Male	226,431	(97)	7,941	(3)	1.00
Female	207,938	(95)	10,913	(5)	1.49 (1.45–1.54)***
**Mother’s education**					
≤9 years	178,468	(95)	8,675	(5)	1.00
Upper secondary	159,993	(97)	5,628	(3)	0.72 (0.69–0.75) ***
University	32,717	(97)	966	(3)	0.61 (0.57–0.65)***
Data missing	63,191	(95)	3,585	(5)	

**Table 2:** The adjusted associations between perinatal factors and receiving a DP.

Perinatal factors	Multivariable results		
	Model 1: (n = 322,464) AOR (95% CI) p-value	Model 2: (n = 273,708) AOR (95% CI) p-value	Model 3: (n = 273,708) AOR (95% CI) p-value
**Birth defect**			
No	1.00	1.00	1.00
Yes	2.31 (2.18–2.45) ***	2.52 (2.36–2.69) ***	2.74 (2.49–3.00)***
**Apgar score**			
7–-10	1.00	1.00	1.00
<7	2.12 (1.91–2.35)***	2.19 (1.95–2.47)***	2.12 (1.77–2.52)***
**Small for gestational age (SGA)**			
No	1.00	1.00	1.00
Yes	1.84 (1.72–1.96) ***	1.73 (1.61–1.86) ***	1.73 (1.54–1.94)***
**Sex**			
Male		1.00	1.00
Female		1.54 (1.48–1.59)***	1.55 (1.46–1.64)***
**Mother’s education**			
≤<9 years		1.00	1.00
Upper secondary		0.76 (0.73–0.79)***	0.74 (0.69–0.79)***
University		0.63 (0.58–0.68)***	0.67 (0.59–0.75)***
Birth defect*sex			
Male: No birth defect			1.00
Female: birth defect			0.85 (0.75–0.97)*
Sex*Apgar score			
Male: Apgar 7–-10			1.00
Female: Apgar <7			1.07 (0.84–1.35)
Sex*Small for gestational age (SGA)			
Male: not SGA			1.00
Female: SGA			1.00 (0.86–1.16)
Mother’s education*sex			
Male: <9 years			1.00
Female: Upper secondary			1.04 (0.96–1.13)
Female: University			0.90 (0.77–1.06)
Birth defect*Apgar score			
Birth defect No*Apgar score 7–10			1.00
Birth defect Yes*Apgar score <7			1.26 (0.89–1.76)
**Birth defect** *SGA			
Birth defect No*SGA No			1.00
Birth defect Yes*SGA Yes			1.39 (1.12–1.74)*
SGA*Apgar score			
SGA No*Apgar score 7–-10			1.00
SG Yes*Apgar score <7			0.86 (0.63–1.16)

AOR-Adjusted odds ratio; Model 1 contains perinatal health variables, Model 2 adds sex and mother’s education level to Model 1, and Model 3 extends Model 2 by including interaction effects.

*** p-value <0.001

* p-value <0.05

Furthermore, we performed stratified logistic regression analyses to account for the different ages at the beginning of receiving a DP (Table 3). We created separate models for ages 16–18, 19–29, and 30–33. In each of these models, we used the same set of covariates and interaction terms as those used in Model 3. Model 4 estimated the odds of receiving a DP at ages 16–18. Model 5 assessed the odds of receiving a DP at ages 19–29 given that they did not receive a DP earlier. Model 6 assessed the odds of receiving a DP at ages 30–33 among those who started on a DP at this age (Table 3). We assessed multi-collinearity for all adjusted models by calculating the variance inflation factor and regressing each independent variable on all the other independent variables, and we found no strong evidence for multi-collinearity. Using the likelihood ratio test, we evaluated the overall fitness of the models. Odds ratios (ORs) with 95% confidence intervals (CIs) were reported, and statistical significance was set at p < 0.05. We performed all statistical analyses using the R software package.

**Table 3:** Shows the associations between perinatal factors and receiving a DP in the 1973–1977 birth cohort, stratified by age at the start of receiving a DP and adding all possible interactions.

	Model 4: Age 16–18 years AOR (95% CI) p-value	Model 5: 19–29 years AOR (95% CI) p-value	Model 6: 30–33 years AOR (95% CI) p-value
**Birth defect**			
No	1.00	1.00	1.00
Yes	5.89 (5.06–6.84)***	1.49 (1.24–1.79)***	1.39 (1.08–1.78)**
**Apgar score**			
7–10	1.00	1.00	1.00
<7	4.25 (3.12–5.66)***	1.53 (1.41–1.67)*	1.25 (0.75–1.97)
**Small for gestational age (SGA)**			
No	1.00	1.00	1.00
Yes	2.15 (1.69–2.69)**	1.49 (1.22–1.80)***	1.43 (1.09–1.84)***
**Sex**			
Male	1.00	1.00	1.00
Female	0.73 (0.64–0.85)***	1.53 (1.41–1.67)***	2.16 (1.95–2.40) ***
**Mother’s education**			
≤9 years	1.00	1.00	1.00
Upper secondary	0.75 (0.66–0.85)***	0.71 (0.65–0.79)***	0.72 (0.64–0.82)***
University	0.76 (0.59–0.96)*	0.66 (0.54–0.79)***	0.56 (0.43–0.73)***
**Interaction terms**			
Sex*Birth defect			
Male: No birth defect	1.00	1.00	1.00
Female: birth defect	1.27 (1.03–1.58)*	0.99 (0.78–1.26)	0.88 (0.64–1.21)
Sex*Apgar score			
Male: Apgar 7–10	1.0	1.00	1.0
Female: Apgar <7	1.22 (0.82–1.80)	1.46 (0.98–2.20)	0.85 (0.47–1.55)
Sex*Small for Gestational age			
Male: not SGA	1.00	1.00	1.00
Female: SGA	1.39 (1.04–1.86)**	1.02 (0.81–1.30)	0.98 (0.72–1.35)
Sex*Mother’s education			
Female: ≤9 years	1.00	1.00	1.00
Female: Upper secondary	1.21 (0.99–1.47)	1.11 (0.98–1.25)	0.97 (0.83–1.14)
Female: University	1.25 (0.88–1.77)	0.93 (0.73–1.19)	0.84 (0.59–1.18)
Birth defect*Apgar score			
Birth defect No*Apgar score 7–10	1.00	1.00	1.00
Birth defect Yes*Apgar score <7	0.90 (0.57–1.40)	0.86 (0.39–1.67)	1.07 (0.31–2.76)
**Birth defect** *SGA			
Birth defect No*SGA No	1.00	1.00	1.00
Birth defect Yes*SGA Yes	1.15 (0.83–1.58)	1.27 (0.82–1.89)	1.06 (0.55–1.86)
SGA*Apgar score			
SGA No*Apgar score 7–10	1.00	1.00	1.00
SGA Yes*Apgar score <7	0.56 (0.34–0.91)*	0.84 (0.49–1.37)	1.29 (0.61–2.52)

Models 4–6 contain all the studied perinatal variables, showing the main effect and interaction effects.

*** p-value < 0.001

** p-value < 0.01 and

* p-value < 0.05

### Ethical considerations

Statistics Sweden made the data anonymous therefore obtaining the informed consent for each individual was neither necessary nor possible with anonymous data. The Regional Ethical Vetting Board approved this research based on data in the Umeå SIMSAM Lab (Dnr 2010-157-31 Ö).

## Results

We present the differences in the study population’s characteristics in Table 1. The total number of people who began receiving a DP between the ages of 16 and 37 years during the follow-up period was 18,854 (4% of the 453,223 participants). Of these 4,007 (21%) received a DP at 16–18 years of age, 8,292 (44%) at 19–29 years, 5,425 (29%) at 30–33 years, and 1,133 (21%) at 34–37 years. The proportion of individuals with birth defects who received a DP was twice as large when compared to individuals without birth defects (8% vs. 4%, respectively). Receiving a DP was more common among females than males, 5% versus 3%, respectively. The prevalence of DP was highest among those with maternal education ≤9 years of schooling (5%), but the prevalence was similar among those whose mothers had upper secondary or university education (3% for both) (Table 1).

The bivariate results showed that persons with a birth defect were at higher odds of receiving a DP compared to persons without a birth defect. Those with low Apgar scores were more likely to receive a DP compared to those with high Apgar scores, and persons who were small for gestational age had increased odds of receiving a DP compared to those who were not. Women had increased odds of receiving a DP than men, and high maternal education level was associated with reduced odds of receiving a DP (Table 1).

In Table 2, simultaneously adjusting for the three perinatal health variables in Model 1 confirmed the significantly increased odds of receiving a DP among persons with birth defects, a low Apgar score, and being small for gestational age. In Model 2, birth defects, low Apgar score, small for gestational age, and being a woman remained significantly associated with higher odds of receiving a DP, while high maternal education was significantly associated with lower odds of receiving a DP. In Model 3, we added interaction terms to the previous model (Model 2), and the main effect remained significant and in a similar direction as observed in the previous model. We found significant interactions between birth defect and sex and between birth defect and small for gestational age, while the other interaction effects were not statistically significant (Table 2). The likelihood ratio test for the fitness of the models found significant evidence for the overall effects of all independent variables on the dependent variable.

In Table 3, we present the association between perinatal conditions and the odds of receiving a DP in the three different age groups. Among those who started receiving a DP between the ages of 16 and 18 years (Model 4), the new recipients were significantly more likely to have had a birth defect, a low Apgar score, or been small for gestational age. In this youngest age group, we found evidence of interactions; women who had a birth defect were at higher odds of receiving a DP compared to men who had birth defects and women born small for gestational age were more likely to receive a DP compared to men born small for gestational age. Persons born small for gestational age and having low Apgar score were less likely to receive a DP. Model 5 shows the increased odds of receiving a DP between the ages of 19 and 29 among those who had a birth defect, a low Apgar score, or who were small for gestational age, and women. In Model 6, starting to receive a DP at the age of 30–33 was associated with having had a birth defect and being small for gestational age, but not with a low Apgar score. High maternal education was associated with lower odds of receiving a DP for all age groups presented in Table 3. We found no interaction effect for the age groups 19–29 or 30–33 years. (Table 3).

## Discussion

Our findings support the study’s main hypothesis that having a birth defect is significantly associated with beginning to receive a DP during early adulthood. Having a birth effect was by far the strongest predictor of future use of DP, and this effect was stronger among those who started their DP at age 16–18 years. A low Apgar score was associated with receiving a DP before age 30, but not afterwards. Being small for gestational age was associated with increased risk of receiving a DP. Women were less likely to receive a DP between ages 16 and 18, but had increased odds of receiving a DP from age 19 onwards. Individuals with higher maternal education level were significantly less likely to receive a DP as age increased. We further observed that the main effect of the studied perinatal health indicators was strongest among those who started to receive a DP at 16–18 years, but the strength of the association reduced as the age when beginning to receive a DP increased, even though this effect remained statistically significant.

As far as we are aware, ours is the first study to show the associations between birth defects, Apgar score at 5 minutes, and receiving a DP at ages 16–37. These findings confirm our hypothesis that having a birth defect and low Apgar score is associated with increased odds of receiving a DP early in life. These observations are biologically plausible and support existing literature reporting a link between birth defects, [21, 23] low Apgar score at 5 minutes, and other forms disability such as a developmental disability. [25, 26] Our finding that individuals who were small for gestational age were more likely to receive a DP during early adulthood supports earlier studies that report similar associations. [11, 19, 20] The reasons for the increased odds of receiving a DP among women was partly explained by having a birth defects and being born small for gestational age.

We report that the effects of the perinatal health factors appeared to weaken as age at the start of the DP increased. This might imply that individuals who had severe health problems needed to start receiving a DP earlier. It could also be that as these individuals get older the effects of adulthood exposures such as socio-demographic factors become more pronounced [6–12], outweighing the effects of the perinatal health factors. Future studies extending the model to include adult factors could help clarify this association.

The fact that higher odds of receiving a DP were associated with low maternal education concurs with the theory of fetal origin. Low maternal education possibly indicates a poor socio-economic position before or immediately after birth, predisposing offspring to further socio-economic, behavioral, and pathological disadvantages across the lifespan [16, 19]. Our finding that women were more likely to receive a DP is in line with reports from previous studies in Sweden [1, 2] and from other European countries. [31]. Several previous studies have also reported gender differences in the use of DP. [1, 6, 20, 31, 32] We found that women had lower odds of receiving a DP at ages 16–18, but had higher odds from age 19 and onwards. A previous study reported an increase in women’s DP incidence rates beginning in their late twenties and onwards, but not before this age. [20] The explanation for these gender differences is not clear, but previous research has attributed them to the underlying structure that dictates gendered living and working conditions, exposing women engaging in paid work to a “double burden” that results from combining work with responsibility for the home and the children. As a result, women’s health tends to suffer as they reach the age that requires combining gainful employment with family life. [33, 34] We also observed that in the youngest age group the effect of birth defect and small for gestational age on DP differed by sex. The mechanisms behind the observed sex difference are not clear, but might be due to other health and socio-economic conditions outside the scope of this study.

### Study strengths and weaknesses

The strength of our study stems mainly from using register data with nationwide coverage, which ensured high completeness, limited follow-up loss, and no recall bias. This study suffers from some limitations, such as a potential selection bias relating to death as an exclusion criterion. Children with adverse perinatal health outcomes are most likely to die, and hence the exclusion of children who died might have led to underestimations of the effect, and potentially a selection bias. However, we still observed high odds among those with adverse health indicators. Our results reflect the situation among those who were alive from ages 16 to 37. The change in the minimum age eligibility for the DP, from age 16 to age 19 in 2002, might have led to classification bias in our study. However, we consider this a minor problem because the eligibility criteria remained based on the presence of a long-term work-disabling health condition, both prior to and beyond 2002. Additionally, we considered the legislation change by analyzing data for the different age groups separately, i.e. ages 16–18, 19–29, and 30–33. We also restricted this age-stratified analysis to persons up to age 33 to make the groups as comparable as possible.

## Conclusion

This study showed that negative perinatal health outcomes as measured by having birth defects, low Apgar score at 5 minutes, and being small for gestational age were associated with increased odds of receiving a DP during early adulthood. Moreover, persons who had negative perinatal outcomes started on DP at a very early age (16–18 years). Our findings support a continued need to promote perinatal health, as this might ensure continued favorable health and less need for DP in early adulthood. The fact that women and persons with low maternal education were more likely to receive a DP suggests a need for a continued review of public policies aimed at reducing gender and social inequalities. Future studies should attempt to explain pathways between perinatal health and DP and to understand the disparities in DP by mother’s education, sex, and age.
